# The enduring effects of adverse childhood experiences (ACES) on mood dysregulation in children: A literature review

**DOI:** 10.1192/j.eurpsy.2021.561

**Published:** 2021-08-13

**Authors:** F. Arain, N. Chavannes, C.C. Corona, A. Tohid, H. Arain, M. Jennings, A. Sanchez-Lacay

**Affiliations:** 1 Psychiatry, BronxCare Health System Mount Sinai, NY, United States of America; 2 Psychiatry, University of Southern California, Los Angeles, United States of America; 3 Under Graduate, Columbia University, New York, United States of America

**Keywords:** Adverse childhood experiences, juvenile offenders, prevention

## Abstract

**Introduction:**

Behavioral dysregulation is a common presentation of children in the Emergency-Room (ER)^1^. A 10-year-old African-American boy with attention-deficit/hyperactivity disorder, oppositional defiant disorder with poor treatment adherence, two previous psychiatric hospitalizations and multiple ER visits, presented with dysregulation and aggressive behavior. He had inconsistent parenting and poor attachment with present involvement of child protective services. We did a systematic review to interpret associations between adverse childhood experiences (ACEs) and the development of behavioral dysregulation in later life.

**Objectives:**

To see associations between ACEs and the development of behavioral dysregulation in later life.

**Methods:**

We searched PsycINFO, APA PsycNet, PubMed, and Medline. Among 35 articles, five were included: 1) a meta-analysis of health consequences and ACEs^1^; 2) a data analysis of 64,329 youth from the Florida Department of Juvenile Justice that focused on suicide attempts and ACEs^2^; 3) a systematic review of 42 articles related to ACEs ^3^; 4) data from 22,575 youth for childhood abuse, trauma and neglect ^4^ and 5) a multimodal logistic regression study on 64,000 juvenile offenders focused on ACE scores and latent trajectory.^5^

**Results:**

There is increased risk of substance use, mental and physical health problems, and violence associated with ACEs^1, 2^. The relationship between childhood difficulties and suicide is interceded by adolescent’s maladaptive behaviors^3^. By age 35, ACEs increase the risk of becoming a serious juvenile offender^4^. Increased exposure to ACEs differentiates early-onset and sustained criminality from other forms of criminality^5^.

**Conclusions:**

ACEs can affect the development of a child in multiple ways including suicidal behavior, aggression, impulsivity, criminality, academic difficulties and substance abuse

